# Meta-Analysis-Based Preliminary Exploration of the Connection between ATDILI and Schizophrenia by *GSTM1/T1* Gene Polymorphisms

**DOI:** 10.1371/journal.pone.0128643

**Published:** 2015-06-05

**Authors:** Lei Cai, Mei-Hong Cai, Mei-Yan Wang, Yi-Feng Xu, Wen-Zhong Chen, Shen-Ying Qin, Chun-Ling Wan, Lin He

**Affiliations:** 1 Bio-X Institutes, Key Laboratory for the Genetics of Developmental and Neuropsychiatric Disorders (Ministry of Education), Shanghai Jiaotong University, Shanghai, 200030, China; 2 Shanghai Mental Health Center, Shanghai Jiaotong University, Shanghai, 200240, China; 3 School of Medicine, Shanghai Jiaotong University, Shanghai, 200020, China; 4 School of Life Sciences and Technology, Tongji University, Shanghai, 200092, China; UTHSCSH, UNITED STATES

## Abstract

Anti-tuberculosis drugs have some adverse effects such as anti-tuberculosis drug-induced liver injury (ATDILI) and mental disorders. The involvement of glutathione S-transferase (GST) genes in pathogenesis of ATDILI or schizophrenia (SCZ) has been reported. Therefore, *GST* genes may exemplify molecular connectors between ATDILI and SCZ. However, association studies of *GSTM1/T1* polymorphisms with these two diseases have yielded conflicting results. After searching case-control association studies in PubMed, ISI Web of Science, EMBASE, Chinese National Knowledge Infrastructure (CNKI), and Chinese BioMedical Literature Database, we performed meta-analyses across a total of 20 published association studies on 3146 subjects for the association of *GSTM1* and ATDILI, 2587 for the *GSTT1*-ATDILI association, 2283 for *GSTM1*-SCZ and 1116 for *GSTT1*-SCZ to test the associations of *GSTM1/T1* polymorphisms with ATDILI and SCZ. The *GSTM1* present genotype was significantly associated with decreased risks of ATDILI (risk ratio(RR): 0.81, 95% confidence interval (CI): 0.75–0.88, *P* < 0.0001) and SCZ (RR: 0.88, 95%CI: 0.80–0.96, *P* = 0.004) according to the fixed-effect model, while the GSTT1 present genotype was significantly associated only with a high risk of SCZ (RR: 1.17, 95%CI: 1.04–1.32, *P* = 0.01) according to both the random- and fixed-effect models, but not with ATDILI (*P* = 0.82) according to the fixed-effect model. Moreover, these significant results were supported with moderate evidence according to the Venice criteria. These results indicate that *GSTM1* represents a genetic connection between ATDILI and SCZ, and suggest that ATDILI and SCZ may be co-occurring for the subjects with *GSTM1* null genotype.

## Introduction

Tuberculosis (TB) remains a devastating disease and the major leading cause of death worldwide. In 2011, an estimated 8.7 million new TB cases were reported, and 1.4 million people died from TB [1]. First-line therapeutic agents, such as isoniazid (INH), rifampin (RIF) and pyrazinamide (PZA) are effective treatments for TB [2]. However, these drugs can induce various adverse effects, among which anti-tuberculosis drug-induced liver injury (ATDILI) is the most common and serious side effect [3–5]. ATDILI, caused by the drugs’ reactive metabolites instead of their direct toxicities, has been widely suggested to be a Glutathione S-transferases (*GSTs*) related disease [6–8].

Schizophrenia (SCZ) is a severe, disabling and chronic mental disorder affecting approximately 1% of the general population [9,10]. Oxidative metabolite damage to neuronal cells is considered to be one of the risk factors for the development of Schizophrenia [11]. Several lines of evidence have suggested that GSTs can modulate the progress of SCZ, given that GSTs and glutathione-related enzymes can detoxify oxidative damage products [12–14]. Thus, *GST* genes are hypothesized to play an important role in both ATDILI and SCZ, acting as ‘molecular bridges’ between these two diseases.

As phase II detoxification enzymes, GSTs have important protective effects of detoxification of anti-TB drugs’ reactive metabolites and products of oxidative stress through conjugating glutathione with target toxic substances and facilitating their elimination from the body [12,15]. GSTs comprise a superfamily of ubiquitous, multifunctional enzymes with two main genes being the glutathione S-transferase Mu-1 (*GSTM1*) gene, located on chromosome 1p13.3,which codes for cytosolic GST class Mu 1 enzyme, and the glutathione S-transferase theta-1 (*GSTT1*) gene, located on chromosome 22q11.2, which codes for cytosolic GST class theta 1 enzyme [16,17]. Both *GSTM1* and *GSTT1* have a null mutation consisting of the complete deletion of the respective gene through homologous unequal crossing over [18–20]. Homozygous null mutations of *GSTM1* and *GSTT1* gene can cause the absence of GST activity, and thus may induce different diseases, including ATDILI and SCZ. However, so far the studies testing the association between *GSTM1/T1* polymorphisms and SCZ or ATDILI have produced conflicting results [21–23].

To assess whether the *GST* genes represent ‘molecular connectors’ between ATDILI and SCZ, we have performed meta-analyses of published studies that test the association between *GSTM1/T1* polymorphisms and ATDILI or SCZ.

## Materials and Methods

### Literature search strategy

The digital medical databases PubMed, ISI Web of Science, EMBASE, Chinese National Knowledge Infrastructure (CNKI) and Chinese BioMedical Literature Database were searched for studies with publication date up to Nov. 30 2013 using the following keywords: (‘anti-tuberculosis drug-induced liver injury’, ‘anti-tuberculosis drug-induced hepatotoxicity’, ‘ATDILI’ or ‘ATDILI’) and (‘Schizophrenia’), respectively, combined with (‘glutathione S-transferase’, ‘*GST*’, ‘*GSTM*’, ‘*GSTM1*’, ‘*GSTT*’, or ‘*GSTT1*’). Furthermore, references of retrieved articles were also reviewed for the literature that they cite.

### Inclusion and exclusion criteria

Articles included in the meta-analysis complied with the following criteria: 1) original case-control association studies with complete data were based on unrelated, randomly selected individuals; 2) cases were TB patients with ATDILI and controls were TB patients without ATDILI, or cases were Schizophrenic patients and controls were healthy subjects; 3) both cases and controls were matched for sex and age; 4) association studies of GSTM1/T1 polymorphisms with SCZ or ATDILI. Other studies, such as: case-only studies, duplications, animal studies, comparisons of laboratory methods, editorials, and review articles were excluded.

### Data extraction

Data extraction was performed independently by two reviewers using a standardized protocol and reporting form. The discrepancy between the two reviewers was resolved by further discussion with a third party. For overlapping studies, the study with the larger sample size was retained for the meta-analysis. The recorded study characteristics included: 1) first author’s name; 2) publication year; 3) sample ethnicity; 4) number of cases and controls for GSTM1/T1 null and present genotypes; 5) control and case characteristics; 6) sex proportion; 7) mean age of cases and controls; 8) methods used for genotyping; and 9) PCR primers and amplification regions.

### Statistical analysis

The strengths of the associations between *GSTM1* and *GSTT1* polymorphisms and the risks of ATDILI or SCZ were measured using risk ratios (RRs) with corresponding 95% confidence intervals (CIs)[24]. Pooled RRs were calculated for null vs. non-null (present) genotypes. Two models of meta-analysis for calculating the pooled RRs were applied, the random-effect model and the fixed-effect model, using the DerSimonian–Laird and Mantel-Haenszel methods, respectively [25,26]. The former assumes that the study samples are taken from populations with varying effect sizes, calculating the study weights both from within-study and between-study variances, while the latter assumes that the study samples are drawn from populations with the same effect size, making an adjustment to the study weights on the basis of the within-study variance [27]. Between-study heterogeneity was assessed with the Chi-square-based Q-test (Cochran’s Q statistic), and P <0.1 was considered statistically significant [28]. The *I*² statistic was also calculated to quantify the proportion of the total variation due to heterogeneity, and *I*² >50% was considered to be statistically significant [29]. If the P value of the heterogeneity test was > 0.1, pooled RRs were evaluated according to the fixed-effect model. Otherwise, the pooled RRs were calculated according to the random-effect model. Subgroup analyses were performed to evaluate racial effects. Power analysis was performed using the Power and Sample Size Program with α = 0.05 as the level of significance and the effects sizes estimated from the meta-analyses [24]. A sensitivity analysis in which one study at a time was removed and the remainder analyzed was conducted to evaluate whether the results could have been affected significantly by a single study. Publication bias was assessed through the Egger weighted regression method and Begg’s test, and P < 0.05 was considered representative of statistically significant publication bias [30]. All statistical analyses were performed using Review Manger 5.2 (The Nordic Cochrane Centre, Copenhagen, Denmark) and Stata version 11.2 (Stata Corporation, College Station, TX, USA).

### Credibility of meta-analysis results

The cumulative evidence for the genetic association of GSTM1 and GSTT1 present genotypes with ATDILI/SCZ, respectively, was assessed according to the Venice interim criteria including amount of evidence, replication of results and protection from bias [31]. With regard to assessment of amount of evidence, grade A was given for n_minor_ >1,000, grade B for 100≤n_minor_≤1,000 and grade C for n_minor_ <100. Here, the n_minor_ referred to the total number of cases and controls with the least frequent genotype. To assessment of replication, grade A was given for *I²*<25%, grade B for 25%≤*I²*≤50% and grade C for *I²*>50%. To assess bias protection, any of following criteria were required: 1) clear phenotype definition; 2) high genotyping quality with a low genotyping error rate; 3) no loss of significance when the first published study was excluded; and 4) no evidence of small-study effects based on a Harbord regression test (significance, *P*<0.05) [32].

## Results

### Characteristics of the included studies

**Fig 1:**
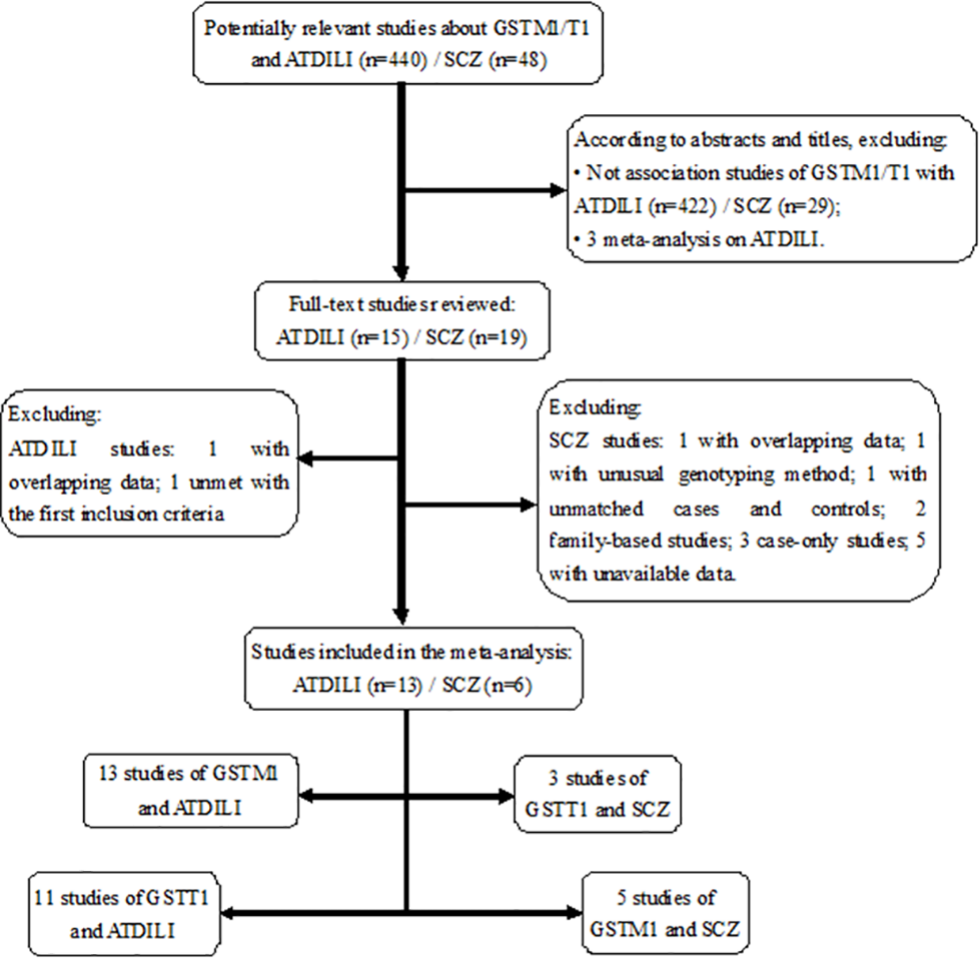
A flow diagram of the study selection process.

A flow diagram summarizing the study selection process was shown in Fig 1. A total of 15 and 19 potentially relevant studies on the associations between *GSTM1/T1* polymorphisms and respective risk of ATDILI and SCZ, respectively, were identified after firstly screening based on the titles and abstracts of the candidate articles. After the second screening, totally, there were 965 cases and 1881 controls in 13 studies of *GSTM1* and ATDILI, 811 cases and 1476 controls in 11 studies of *GSTT1* and ATDILI, 1101 cases and 1182 controls in 5 studies of *GSTM1* and SCZ, and 568 cases and 548 controls in 3 studies of *GSTT1* and SCZ. The detailed characteristics of each study are listed in S1 Table. All these studies were confirmed to relate to the same complete loss of gene mutation of *GSTM1* or *GSTT1*. The genotype distributions across all studies for cases and controls of ATDILI and SCZ are shown in Table 1 and Table 2, respectively.

**Table 1:** Genotype distributions of GSTM1/T1 polymorphisms among ATDILI as cases and non-ATDILI as controls.

First author, Publication year	Race	Cases			Controls		
		Null	Present	Total	Null	Present	Total
An, 2010	East Asian	64	37	101	55	52	107
Chatterjee, 2009	Caucasian	25	26	51	49	51	100
Guo, 2009	East Asian	50	56	106	25	81	106
Huang, 2007	East Asian	42	21	63	29	34	63
Leiro, 2008	Caucasian	12	23	35	25	35	60
Monteiro, 2012	Caucasian	21	38	59	34	84	118
Roy, 2001	Caucasian	17	16	33	8	25	33
Sotsuka, 2011	East Asian	12	8	20	50	42	92
Tang, 2012	East Asian	55	34	89	203	153	356
Teixeira, 2011	Caucasian	11	15	26	61	80	141
Wang, 2010	East Asian	63	41	104	54	57	111
Zhu, 2011	East Asian	133	95	228	152	148	300
Zhu, 2004	East Asian	21	29	50	53	241	294
An, 2010	East Asian	48	53	101	49	58	107
Chatterjee, 2009	Caucasian	3	48	51	3	97	100
Guo, 2009	East Asian	53	53	106	44	62	106
Huang, 2007	East Asian	24	39	63	25	38	63
Leiro, 2008	Caucasian	17	18	35	16	44	60
Monteiro, 2012	Caucasian	11	48	59	28	90	118
Roy, 2001	Caucasian	5	28	33	1	32	33
Sotsuka, 2011	East Asian	7	13	20	40	52	92
Tang, 2012	East Asian	40	49	89	164	192	356
Teixeira, 2011	Caucasian	4	22	26	27	114	141
Zhu, 2011	East Asian	103	125	228	148	152	300

**Table 2:** Genotype distributions of GSTM1/T1 polymorphisms among SCZ and healthy control.

First author, Publication year	Race	Cases			Controls		
		Null	Present	Total	Null	Present	Total
*GSTM1* polymorphism							
Gravina, 2011	Caucasian	82	56	138	70	63	133
Harada, 2001	East Asian	57	30	87	87	89	176
Pae, 2004	East Asian	70	41	111	61	69	130
Raffa, 2013	Caucasian	79	59	138	63	60	123
Watanabe, 2010	East Asian	339	288	627	322	298	620
*GSTT1* polymorphism							
Gravina, 2011	Caucasian	25	113	138	30	103	133
Raffa, 2013	Caucasian	59	79	138	67	56	123
Saadat, 2007	Caucasian	52	240	292	99	193	292

### Association of *GST* polymorphisms with ATDILI

**Fig 2:**
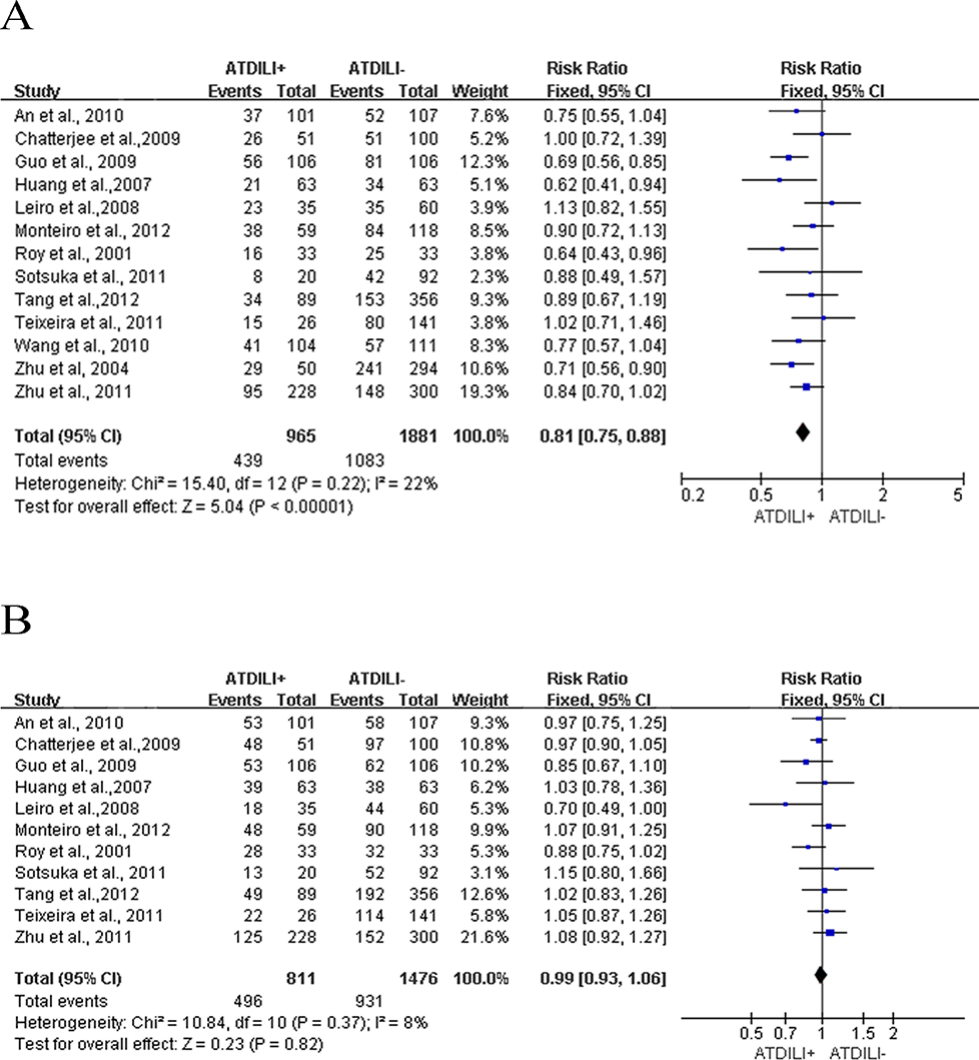
Forest plots for meta-analysis of GSTM1/T1 polymorphisms and ATDILI. A. the summary of RRs with 95% CIs for GSTM1 present genotype; B. the summary of RRs with 95% CIs for GSTT1 present genotype.

The evaluation of the associations between *GSTM1/T1* polymorphisms and risk of ATDILI is summarized in Fig 2. Because no significant heterogeneity was found in either analysis (P = 0.22 and *I* ^*2*^ = 22% for *GSTM1*, P = 0.37 and *I* ^*2*^ = 8% for *GSTT1*), the fixed-effect model was used to analyze both *GSTM1* and *GSTT1*. The frequencies of both *GSTM1* and *GSTT1* null genotype were higher among cases than among controls (54.51% vs. 42.42% for *GSTM1* and 38.84% vs. 36.92% for *GSTT1*). This finding indicated that the *GSTM1* present genotype was significantly associated with a decreased risk of ATDILI (RR: 0.81, 95%CI: 0.75–0.88, P < 0.0001) (Fig 2A), whereas no significant association was found between the *GSTT1* present genotype and ATDILI (RR: 0.99, 95%CI: 0.93–1.06, P = 0.82) (Fig 2B).

In subgroup analyses of the association between *GSTM1* and *GSTT1* genotypes and ATDILI (S1A and S1B Fig), a significant association was identified between the *GSTM1* present genotype and a decreased risk of ATDILI among an East Asian population under a fixed-effect model (RR: 0.77, 95%CI: 0.70–0.85, P = 0.68 for heterogeneity). The regression model indicated strong evidence for an association between the race and the effects of the *GSTM1* present genotype (*P* = 0.033). However, although the total sample size power for detecting a significant effect between the GSTM1 present genotype and ATDILI exceeded 99%, the sample size power in the East Asian group exceeded 99%, whereas that in the Caucasian group was only 14.8%. Given the low power value in the Caucasian group, we are unable to definitively conclude on the association between GST genes and ATDILI in the Caucasian populations.

### Association of *GST* polymorphisms with SCZ

**Fig 3:**
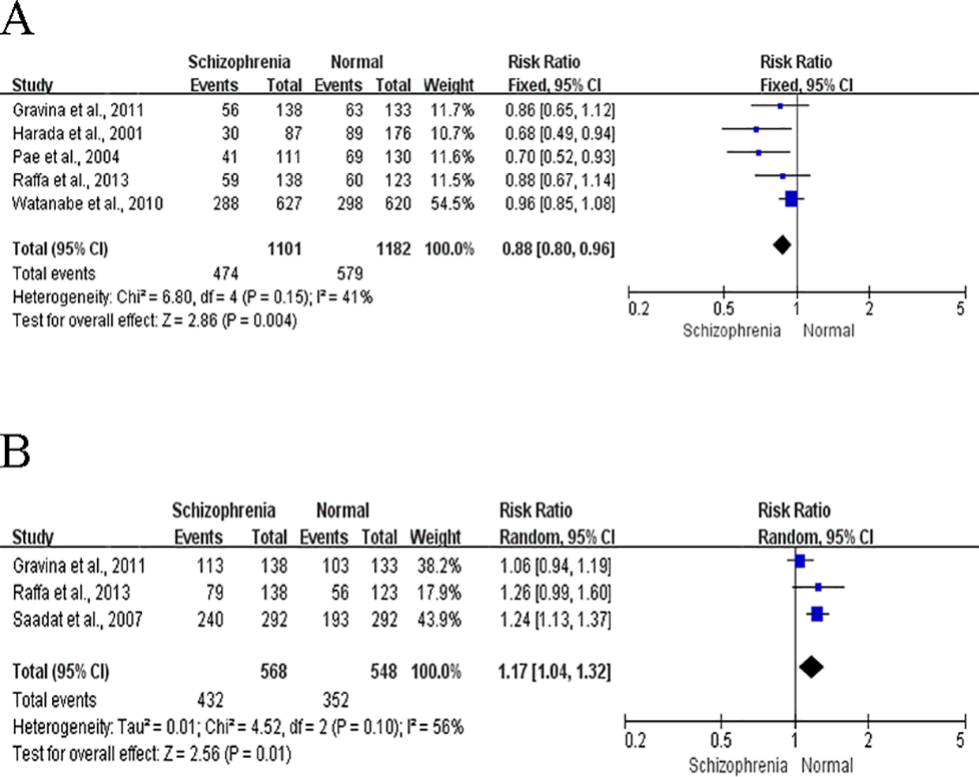
Forest plots for meta-analysis of GSTM1/T1 polymorphisms and SCZ. A. the summary of RRs with 95% CIs for GSTM1 present genotype; B. the summary of RRs with 95% CIs for GSTT1 present genotype.

The combined analysis of the associations between *GSTM1/T1* polymorphisms and risk of SCZ are shown in Fig 3. Because no significant heterogeneity was observed in analysis of *GSTM1* (P = 0.22 and *I* ^*2*^ = 41%), the fixed-effect model was used; because mildly significant heterogeneity was observed in analysis of *GSTT1* (P = 0.10, *I* ^*2*^ = 57%), the random-effect model was used. The *GSTM1* null genotype and *GSTT1* present genotype frequencies were higher among cases than among controls (56.95% vs. 51.02% for *GSTM1* and 23.94% vs. 35.77% for *GSTT1*). This finding indicated that statistically significant associations between the *GSTM1* present genotype and a decreased risk of SCZ (RR: 0.88, 95%CI: 0.80–0.96, P = 0.004) in the fixed-effect model (Fig 3A) as well as between the *GSTT1* present genotype and the risk of SCZ (RR: 1.17, 95%CI: 1.04–1.32, P = 0.01) in the random-effect model (Fig 3B).

Because only three papers concerning the *GSTT1* present genotype were included in the meta-analysis, a subgroup analysis was not performed. No significant associations were found in subgroup analyses of the relationship between the *GSTM1* present genotype with SCZ (S1C Fig). However, the regression model indicated no evidence for an association between race and effects of the *GSTM1* present genotype (*P* = 0.72). The total sample size power for detecting a significant association between *GSTM1* present genotype and SCZ was 83.5%; in contrast, sample size powers were 68.2% for the East Asian group and 32.1% for the Caucasian group.

### Sensitivity analyses and publication bias

A sensitivity analysis via re-analysis after leaving one study out was performed to assess the effect of individual studies on the overall meta-analysis estimate. The *P* values for testing the overall effects after excluding one study ranged from <0.00001 to <0.0001 and from 0.37 to 0.90 in the *GSTM1* and *GSTT1* and ATDILI analyses, respectively; the P values for testing overall effects ranged from 0.0006 to 0.03 in the *GSTM1* and SCZ analyses. These results indicated that the analyses were stable. However, in *GSTT1* and SCZ analysis, the summarized RRs ranged from 1.17 (95%CI: 1.04–1.32) to 1.12 (95%CI: 0.94–1.34) and the P values from 0.01 to 0.20 after excluding a study by Saadat (13); this indicated that this study was the main source of the observed mild heterogeneity. Furthermore, there was no indication of significant publication bias in the overall meta-analysis according to both Egger’s and Begg’s tests (P = 0.766 and 0.951, respectively, for GSTM1 vs. ATDILI; P = 0.137 and 0.35, respectively, for GSTT1 vs. ATDILI; P = 0.066 and 0.221, respectively, for GSTM1 vs.SCZ; and P = 0.114 and 0.296, respectively, for GSTT1 vs. SCZ).

### Credibility of meta-analysis results

In this meta-analysis, strict inclusion criteria were used to address phenotype definitions and genotyping quality. For the meta-analysis of ATDILI studies, the n_minor_ for *GSTM1* null genotype was 1321, indicating a grade of A. The *I* ^*2*^ for the *GSTM1* null genotype was 22%, and a grade of A was given. After excluding study by Roy conducted in 2001 [21], a significant association remained between *GSTM1* present genotype and ATDILI (P<0.0001), and the Harbord test P value was 0.679. For the meta-analysis of SCZ studies, n_minor_ for the *GSTM1* present genotype was 1053, and a grade of A was given. The *I* ^*2*^ for the *GSTM1* null genotype was 41%, resulting in a grade of B. After excluding the study by Harada conducted in 2001[13], a significant association remained between the *GSTM1* present genotype and SCZ (P = 0.03), and the Harbord test P value was 0.0716. Therefore, ‘moderate’ cumulative evidence supported significant associations of the *GSTM1* present genotype with both ATDILI and SCZ.

## Discussion

Previously, ATDILI was recognized as the primary adverse effect of anti-TB drugs. Although mental disorders, of which SCZ is an example, have been considered an additional adverse effect of anti-TB drugs [33], a clear picture of whether there is sharing of common biological determinants among ATDILI and Schizophrenia has yet to emerge. To our knowledge, the current study is the first to explore the molecular connection between SCZ and ATDILI by GST genes via large-scale meta-analyses [28]. We found that the *GSTM1* present genotype was significantly associated with decreased risks of ATDILI (*P* < 0.0001) and SCZ (*P* = 0.004), whereas the *GSTT1* present genotype was only significantly associated with a high risk of SCZ (*P* = 0.01), but not ATDILI (P = 0.82); these significant results were supported by ‘moderate’ evidence according to the Venice criteria. Because a dependent conclusion may have resulted from the evidence that a number of studies simultaneously tested the associations of both *GSTT1* and *GSTM1* with single disorder, a Bonferroni correction method was used to correct the P value. After correction, the *GSTM1* present genotype remained significantly associated with decreased risks of both ATDILI (P < 0.0001) and SCZ (P = 0.008), and the *GSTT1* present genotype remained significantly associated only with a high risk of SCZ (P = 0.02), but not ATDILI (P = 1). These results indicate that *GSTM1*, rather than *GSTT1*, may be a ‘molecular connector’ between ATDILI and SCZ.

GSTs, acting as free radical scavengers through glutathione conjugation to reduce target substances’ potential toxicity, have varied tissue-specific expression patterns [34]. *GSTM1* is mainly expressed in the liver and brain [35], which offers some support for the significant association found here with both ATDILI and SCZ. Furthermore, *GSTM1* is found to not only detoxify anti-TB drugs’ toxic metabolites generated by CYP2E1 in the liver, but also catalyze the conjugation of glutathione with the aminochrome and the dopa-o-quinone metabolite of oxidized dopamine in the brain [36]. In the brain, reactive oxygen species are generated at high rates, and the redox mechanism that controls a balance between neuro destructive oxidants and neuro protective antioxidants partly regulating growth and pruning of neurons [37]. Thus, the inactive *GSTM1* caused by the *GSTM1* null genotype can cause not only liver injury but also accumulation of neuro destructive oxidants leading to Schizophrenia [11]. In contrast, *GSTT1* has considerable presence in the brain, it has only trace expression in the liver [38], and it is not involved in the detoxification of the oxidized o-quinone dopamine metabolite[36]. Furthermore, besides its role as a beneficial scavenger towards electrophiles, mammalian *GSTT1* can act as a deleterious metabolic activator for halogenated compounds to produce a variety of intermediate materials potentially dangerous for DNA and cells [39]. In the current meta-analysis, the results did not show an association between the *GSTT1* gene and ATDILI, but indicated that the *GSTT1* present genotype increases the risk of SCZ.

A meta-analysis with a larger sample size is widely considered to provide greater statistical power to identify the effect of a genetic polymorphism on a disease than a single association study [40]. An assessment of heterogeneity is critical to ensure the credibility of a meta-analysis because studies are pooled based on an assumption of etiological homogeneity across studies. In this meta-analysis, both P value and *I* ^*2*^ were estimated for the heterogeneity test. No significant heterogeneity was observed in the meta-analyses, except for mildly significant heterogeneity in the analysis of *GSTT1* with SCZ. Interestingly, in the subgroup analyses of studies concerning *GSTM1* and ATDILI, a significant association was found in the East Asian group, but not in the Caucasian group. This may be because of the low power value in the latter group (14.8%), which reduces the ability to detect significant effects. Although race was significant associated with the effects *GSTM1* on ATDILI, the biological impact of *GSTM1* gene on the risk of ATDILI may be consistent across racial groups, and this significant result may instead be because of the effects of different cultural and living habits among different races, such as: diet [41]. A sensitivity analysis was also performed to explore the source of the heterogeneity identified in the analysis of *GSTT1* with SCZ and indicated that the study by Saadat greatly affected this analysis; accordingly, these results should be treated with caution. Two meta-analyses have been previously published on *GSTM1/T1* and ATDILI: Cai in 2012 and Tang in 2013 [42,43]. Although the current study yielded similar results, particularly that the *GSTM1* null genotype was significantly associated with an increased risk of ATDILI, a study by Kim was included in those previous meta-analyses, but was excluded from the current analysis[44]. In Kim’s study, the cases with ATD-induced adverse cutaneous reactions are enrolled, and subjects without skin diseases are as controls. We removed this study at the second screening of our study due to its different study design compared to the other studies.

Some potential limitations of our meta-analysis deserve consideration. First, diseases are caused by both genetic and environmental factors; however, we did not include an environmental factor analysis. We attempted to obtain complete information regarding dietary habits and substance abuse, among other factors. However, the collected literature seldom provided data in addition to genotypic data. As a result, the current meta-analysis solely focused on genetic factors. Second, the sample size in the current analysis was limited. Additional association studies focused on the co-morbidities of ATDILI and SCZ, and involving large-scale samples will be required to confirm the current results.

In summary, the current meta-analysis results demonstrate that *GSTM1*, rather than *GSTT1*, may act as a molecular link between ATDILI and SCZ and suggest that ATDILI and SCZ may be concurrent adverse effects of anti-TB drugs in subjects harboring the *GSTM1* null genotype.

## Supporting Information

S1 Fig Forest plots for subgroup meta-analysis of GSTM1/T1 polymorphisms and ATDILI/Schizophrenia. A. the summary of RRs with 95% CIs for GSTM1 present genotype and ATDILI; B. the summary of RRs with 95% CIs for GSTT1 present genotype and ATDILI; C. the summary of RRs with 95% CIs for GSTM1 present genotype and Schizophrenia.
(DOCX)

S1 Table Characteristics of studies included in the meta-analysis. (XLS)
