# Supplementary figures and images for: Meta-Analysis-Based Preliminary Exploration of the Connection between ATDILI and Schizophrenia by GSTM1/T1 Gene Polymorphisms
Source: PLoS One. 2015 Jun 5;10(6):e0128643. doi: 10.1371/journal.pone.0128643 (PMC4457417; doi:10.1371/journal.pone.0128643)

A.
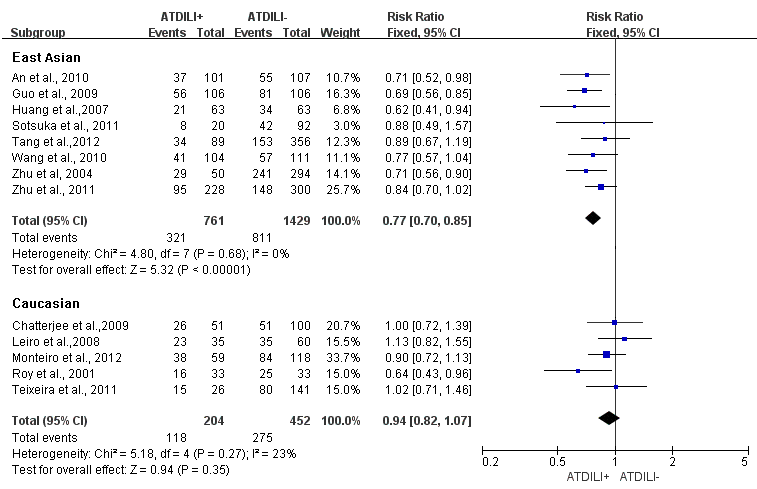


B.
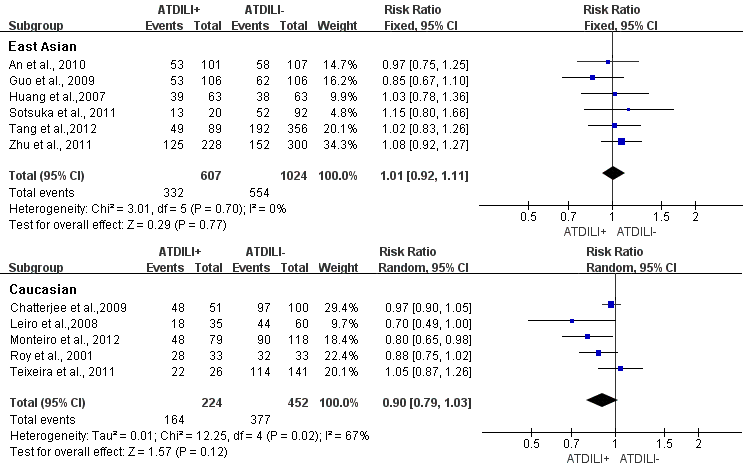


C.
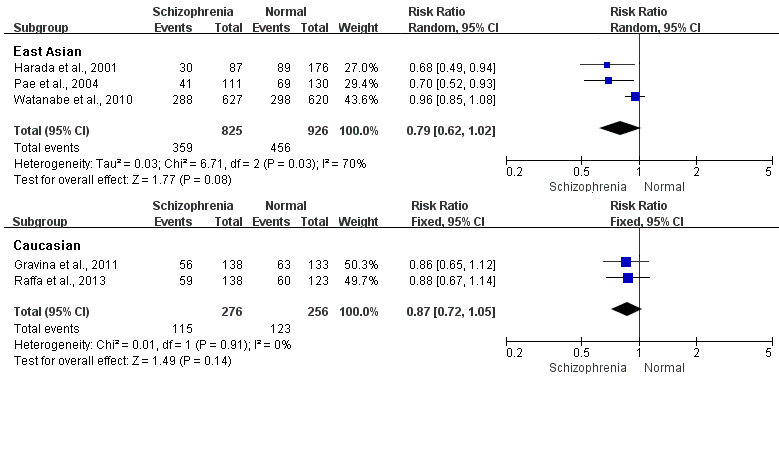


Figure S1

Supplement: S1 Fig — A. the summary of RRs with 95% CIs for GSTM1 present genotype and ATDILI; B. the summary of RRs with 95% CIs for GSTT1 present genotype and ATDILI; C. the summary of RRs with 95% CIs for GSTM1 present genotype and Schizophrenia. (DOCX) [file pone.0128643.s001.docx]
